# Antioxidant Activity of *Radix Cyathula officinalis* Kuan Polysaccharides and Their Modulatory Effects on the Gut Microbiota of *Caenorhabditis elegans*

**DOI:** 10.3390/cimb47070538

**Published:** 2025-07-11

**Authors:** Rui Li, Xinyue Chen, Lijuan Wu, Lei Xie, Mengqiu Chen, Yujie Qiu, Fan Liu, Ji Chen, Mengliang Tian

**Affiliations:** 1 College of Agronomy, Sichuan Agricultural University, Chengdu 611130, China; 71352@sicau.edu.cn (R.L.); chenxinyue@tfswufe.edu.cn (X.C.); wulijuan0921@126.com (L.W.); 17683180748@163.com (L.X.); 19114323880@163.com (M.C.); 80254@sicau.edu.cn (Y.Q.); Liufantl2006@163.com (F.L.); 2 Academy of Agriculture and Forestry Sciences, Qinghai University, Xining 810016, China

**Keywords:** *Radix Cyathula officinalis* Kuan, polysaccharides, antioxidant activity, gut microbiota

## Abstract

Polysaccharides isolated from *Radix Cyathula officinalis* Kuan (RCP) are key bioactive components with immunomodulatory, antioxidant, and anti-inflammatory effects. Their efficacy varies according to their geographic origin and processing methods. However, the systemic anti-aging mechanisms and antioxidant efficacy of RCP have not yet been comprehensively characterized. This study investigated the antioxidant and anti-aging effects of RCP in vitro and in vivo using a *Caenorhabditis elegans* heat stress model, comparing rRCP (RCP from raw samples) and wRCP (RCP from wine-processed samples) from key production areas. Among these, the RCP collected from the Zhonggang region exhibited the strongest antioxidant activity. Both rRCP and wRCP enhanced worms’ oxidative stress resistance, reduced their ROS levels, increased their antioxidant enzyme activities, prolonged their lifespan, and improved their reproductive capacity under thermal stress. Notably, the wRCP exhibited more pronounced benefits. Additionally, 16S rRNA sequencing revealed that RCP altered the gut microbiota’s composition by increasing its microbial diversity, enriching beneficial bacteria like *Bacillus*, and decreasing potential pathogens such as *Escherichia* and *Citricoccus*. The treatment also led to an increased abundance of *Firmicutes* and a slight reduction in *Bacteroidetes*. Collectively, these findings suggest that RCP, particularly wRCP, holds promise as a therapeutic agent for combating oxidative stress and promoting longevity, in part by modulating the gut microbiome.

## 1. Introduction

Oxidative stress has been widely recognized as a pivotal contributor to the onset and progression of numerous diseases, particularly age-associated neurodegenerative diseases [1] such as Alzheimer’s disease [2], Parkinson’s disease [3], and Huntington’s disease [4]. With the accelerating global aging population, the societal and healthcare burdens related to these conditions have intensified. Consequently, promoting healthy aging has attracted increasing attention from clinical, social, and economic perspectives. Interestingly, the intake of antioxidant compounds, either through pharmacological agents or dietary supplements, has emerged as a promising strategy to mitigate oxidative damage, enhance healthspan, and delay the aging process [5]. However, the use of synthetic commercial antioxidants often accompanies undesirable side effects, including hepatotoxicity and potential carcinogenicity [6]. Therefore, the development of natural antioxidants with low or negligible cytotoxicity has become imperative.

Recent studies highlight that plant-derived polysaccharides, particularly those obtained from traditional medicinal herbs, possess potent antioxidant activity [7,8] while exhibiting low cytotoxicity [9]. This underscores their substantial potential as novel candidates for preventing oxidative damage in multifactorial diseases [10,11,12]. For example, *Pueraria lobata* polysaccharides [13] enhance antioxidant capacity, strengthen immune function, improve intestinal barrier integrity, and promote the growth of beneficial gut microbiota while suppressing harmful bacterial proliferation in calves. Similarly, fermented wheat bran polysaccharides [14] act as superior growth promoters in zebrafish by positively regulating intestinal antioxidant-related gene expression and reshaping gut microbial communities. *Spirulina platensis* polysaccharides [15] significantly increase the abundance of beneficial bacteria such as *Achromobacter*, *Pseudoalteromonas*, and *Flavobacterium* in *C. elegans*, while simultaneously reducing malondialdehyde levels, thereby enhancing systemic antioxidant capacity. Moreover, *Cordyceps cicadae* polysaccharides [16] and fucoidan [17] exhibit comparable antioxidant mechanisms, further validating the therapeutic promise of natural polysaccharides in oxidative stress attenuation.

*Cyathula officinalis* Kuan, a perennial herbaceous plant in the family Amaranthaceae, is widely distributed in tropical areas of Asia and Africa, particularly in China (Sichuan, Chongqing, and Hubei Province), Korea, and Vietnam. Its root, known as “Chuan Niu Xi” in Chinese, is officially documented in the Chinese Pharmacopoeia (2020 edition) for its traditional medicinal properties, including promoting blood circulation to remove stasis, regulating menstruation, alleviating joint movement, promoting diuresis, and relieving stranguria [18]. Modern pharmacological studies have identified *Radix Cyathula officinalis* Kuan polysaccharides (RCP) as its primary active constituents, exhibiting significant immunostimulatory [19], antioxidant [20], anti-inflammatory [21], and anti-tumor properties [22]. For example, Han et al. [20] have shown that a water-soluble RCP effectively scavenges free radicals and exhibits antioxidant properties, protecting biological systems from oxidative damage. Li et al. [23] further demonstrated that RCP significantly mitigates oxidative stress induced by D-galactose in animal models, enhancing immune function and metabolic regulation, thereby suggesting its potential as a natural antioxidant source. Despite these findings, studies on the antioxidant potential of RCP remain limited, with most studies confined to in vitro analyses, and few exploring in vivo effects or underlying mechanisms.

Animal models are essential for elucidating the in vivo bioactivities of natural compounds. Compared to traditional models, *Caenorhabditis elegans* offers several unique advantages, including its small and transparent body, rapid life cycle, high progeny numbers, and fully sequenced genome [24]. Importantly, *C. elegans* shares many conserved aging-related genes and pathways with humans, making it an ideal model organism for studying anti-aging substances and mechanisms.

The quality and efficacy of Chinese medicinal materials are influenced by both their geographic origins [25] and processing methods [26]. Notably, wine-processing has been reported to enhance the pharmacological effects of *Radix Cyathula officinalis* Kuan. Currently, raw and wine-processed forms are the two primary clinical preparations of *Radix Cyathula officinalis* Kuan [27]. However, studies on the antioxidant mechanisms of RCP, particularly in its wine-processed form, remain sparse. Thus, this study aimed to collect *Radix Cyathula officinalis* Kuan from different major production regions, analyze and compare the antioxidant activities of crude RCP before and after being wine-processed, and select the samples with the highest antioxidant potential through principal component analysis. Subsequently, we employed a *C. elegans* oxidative stress model to investigate the in vivo antioxidant activities and their effects on gut microbiota, thereby providing a theoretical foundation for the germplasm evaluation and functional exploitation of RCP.

## 2. Materials and Methods

### 2.1. Plant Materials

Three-year-old *Radix Cyathula officinalis* Kuan samples were collected from nine production regions across three provinces in China: Minzhu Village, Yongsheng Township, Jinkouhe District, Leshan City, Sichuan Province (JKH); Wanping Village, Pianma Township, Hanyuan County, Ya’an City, Sichuan Province (HY); Shue Village, Longqiao Township, Fengjie County, Chongqing City (FJ); Hexi Village, Dupi Township, Wushan County, Chongqing City (WS); Maoping Village, Wufeng Town, Wufeng County, Yichang City, Hubei Province (WF); Chaoyang Village, Yanzi Town, Hefeng County, Enshi Prefecture, Hubei Province (HF); Waduanshan Village, Chunmu Camp Township, Lichuan City, Enshi Prefecture, Hubei Province (LC); Zhonggang Village, Longdong Town, Baoxing County, Ya’an City, Sichuan Province (ZG); and Xinkang Village, Fengtongzhai Township, Baoxing County, Ya’an City, Sichuan Province (XK). All samples were authenticated as *Cyathula officinalis* Kuan (Amaranthaceae family). To minimize spatial bias and ensure reproducibility and consistency, samples were collected using the five-point sampling method from five predetermined locations within a defined area: one from the center, and four from equidistant peripheral points arranged in a square around the center. The roots were cleaned, sliced into thin pieces, and dried at 50 °C. One portion of each sample was used as raw material, while the other was soaked in rice wine at a ratio of 10:1 (w/w, root/rice wine), allowed to absorb the liquid, and then stir-fried over a low heat until the surface turned slightly yellow, yielding the wine-processed samples [18]. All samples were ground and sieved through a 40-mesh sieve. This protocol generated 18 powder samples (9 regions × 2 processing methods).

### 2.2. Chemicals and Reagents

Rice wine was purchased from Guyuelongshan Co., Ltd. Anhydrous ethanol, chloroform, n-butanol, anhydrous glucose, concentrated sulfuric acid, ferrous sulfate heptahydrate, cholesterol, magnesium sulfate, calcium chloride, sodium chloride, sodium hypochlorite, and sodium azide were purchased from Cologne Chemical Reagent Co., Ltd. (Chengdu, China). The cellulase enzyme was purchased from Beijing Solarbio Technology Co., Ltd. (Beijing, China), and the catalog number was C8270. The ascorbic acid and catalase (CAT) assay kits were obtained from Beijing Solarbio Technology Co., Ltd. (Beijing, China). The assay kits for the total antioxidant capacity (T-AOC), superoxide dismutase (SOD), reactive oxygen species (ROS), and 2,2′-azino-bis (3-ethylbenzothiazoline-6-sulfonic acid) (ABTS) were purchased from Nanjing Jiancheng Bioengineering Institute (Nanjing, China). The 2,2-Diphenyl-1-picrylhydrazyl (DPPH) was obtained from Beijing Boaotudao Biotechnology Co., Ltd. (Beijing, China). Peptone, tryptone, yeast extract, and agar powder were purchased from Sigma Aldrich (St. Louis, MO, USA). All other chemicals used were of analytical grade.

### 2.3. Preparation of Polysaccharides

In total, 2 g of raw or wine-processed powder was enzymatically hydrolyzed with 7 mg cellulase in 120 mL distilled water at 50 °C for 1 h (with 150 rpm shaking). After enzyme inactivation by boiling for 10 min and ultrasonic extraction (40 kHz, 30 min), the mixture was filtered and centrifuged to collect the supernatant. The supernatant was concentrated to 20 mL at 60 °C, deproteinized three times using the Sevag method, and precipitated overnight with four volumes of 95% ethanol at 4 °C. The precipitate was collected by centrifugation (3600× *g*, 10 min) and subsequently lyophilized to obtain crude rRCP (from the raw sample) and crude wRCP (from the wine-processed sample), respectively. These crude polysaccharides were then dialyzed using a cellulose membrane (MWCO 3.5 kDa) and subsequently lyophilized to yield purified rRCP and wRCP, which were stored at −80 °C for in vivo experiments.

### 2.4. Component Content Analysis

Protein content was measured using the Coomassie Brilliant Blue method [28]. Polyphenol content was determined by the Folin–Ciocalteu method [29]. Polysaccharide content was measured using the phenol–sulfuric acid method [28]. Polysaccharide component fractionation and analysis were conducted following the methodology of Feng Xin et al. [30].

### 2.5. In Vitro Antioxidant Assay

The antioxidant activities of crude RCP were evaluated by measuring free radical scavenging against ABTS, DPPH, and hydroxyl radicals (•OH), as previously described [31]. The total antioxidant capacity (FRAP) was measured using commercial kits following the manufacturers’ instructions. Polysaccharide solutions were prepared at concentrations of 0, 2, 4, 6, 8, 10, and 12 mg/mL in deionized water. All assays were conducted in triplicate.

### 2.6. Cultivation and Synchronization of C. elegans

Wild-type N2 *C. elegans* were obtained from the Caenorhabditis Genetics Center (Minneapolis, MN, USA) and maintained on a nematode growth medium at 20 °C, seeded with *Escherichia coli* OP50. Synchronized L4-stage worms were obtained by allowing egg-laying adults to reproduce and then removing the adults.

### 2.7. In Vivo Antioxidant Assay in C. elegans

#### 2.7.1. Lifespan and Thermal Stress Assay

The synchronized L4-stage nematodes were transferred to nematode growth medium plates containing *Escherichia coli* OP50, 70 mM 5-fluorodeoxyuridine, and various concentrations (0, 0.2, 0.5, 1.0, 1.5, and 2.0 mg/mL) of rRCP or wRCP. The nematodes were subjected to thermal stress at 35 °C, and survival was recorded every 2 h after 4 h of exposure. Lifespan curves were generated, and optimal concentrations were selected based on survival rates. Each treatment was performed in triplicate.

#### 2.7.2. Reproductive Assay

Control worms (CON) were fed *E. coli* OP50; the positive control group was fed OP50 containing 1.5 mg/mL ascorbic acid (AA); and the experimental groups received OP50 containing 1.5 mg/mL of either rRCP or wRCP. Young adult worms were treated at 20 °C for 48 h and then exposed to thermal stress. Ten worms per plate were randomly selected and maintained at 20 °C, with egg-laying recorded every 24 h and worms transferred to fresh plates until reproduction ceased. Total egg production was analyzed to assess potential reproductive toxicity. All treatments were conducted in triplicate.

#### 2.7.3. ROS Detection

After 48 h of rRCP or wRCP treatment at 20 °C, L4-stage nematodes were exposed to 35 °C temperature for 3 h to induce oxidative stress. The nematodes were stained with ROS-sensitive fluorescent probes in the dark for 30 min, anesthetized with sodium azide, and imaged using confocal laser scanning microscopy (Olympus Corporation, Tokyo, Japan). Fluorescence intensity was quantified using ImageJ software 1.8.0.

#### 2.7.4. Antioxidant Enzyme Activity Measurement

Following thermal stress and a 2 h recovery at 20 °C, the worms were collected, washed with a S-basal buffer, and ground in liquid nitrogen. The activities of SOD, CAT, and T-AOC were measured using commercial kits.

#### 2.7.5. Gut Microbiota Analysis

Microbial DNA was extracted using the E.Z.N.A.^®^ Soil DNA Kit (Omega Bio-tek, Norcross, GA, USA) per the manufacturer’s instructions. The V1–V9 regions of bacterial 16S rRNA were amplified using universal primers (27F/1492R), purified, and sequenced using the Illumina MiSeq platform (Shanghai Biozeron Biotechnology Co., Ltd., Shanghai, China). Operational taxonomic units (OTUs) were clustered at 97% similarity using UPARSE, and singletons were removed. Principal coordinate analysis (PCoA) was performed using R software 3.6.3 to analyze gut microbiota composition and diversity.

### 2.8. Statistical Analysis

Statistical analyses were conducted using SPSS software 27.0, and graphs were drawn using GraphPad Prism software 9.5. LEfSe analysis was conducted to identify differentially abundant taxa. A comparison of the data from various groups was performed using one-way ANOVA, and *p* < 0.05 was considered statistically significant.

## 3. Results

### 3.1. Component Composition and In Vitro Antioxidant Activity of Crude RCP

#### 3.1.1. Component Composition of Crude RCP

**Figure 1:**
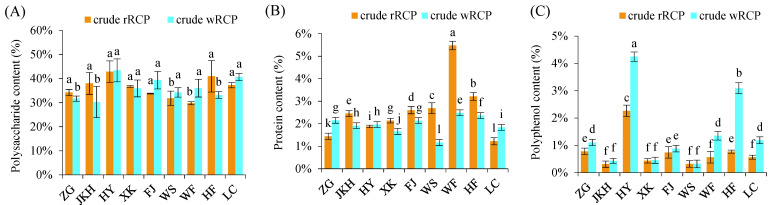
Composition of crude RCP. (**A**) Polysaccharide content of crude RCP; (**B**) protein content of crude RCP; (**C**) polyphenol content of crude RCP. Different letters (a–l) above bars indicate statistically significant differences at *p* < 0.05 level.

The polysaccharide content in crude rRCP from different regions ranged from 29.77% to 42.90%, with HY exhibiting the highest proportion. The ranking, from highest to lowest, was HY > HF > JKH > LC > XK > ZG > FJ > WS > WF. Significant differences in polysaccharide contents were observed between WF, WS, and other regions. In crude wRCP, polysaccharide content ranged from 30.26% to 43.42%, peaking again in HY. The descending order was HY > LC > FJ > WF > XK > WS > HF > ZG > JKH. Significant differences were observed between JKH, ZG, HF, and the other regions (Figure 1A). The protein contents in crude rRCP varied from 1.22% to 5.48% across regions, with WF exhibiting the highest content. Significant differences were noted among all regions. In crude wRCP, protein content ranged from 1.17% to 2.49%, with WF remaining the dominant source. Differences were significant among most regions, except for some comparisons between ZG and FJ, and JKH and HY (Figure 1B). The polyphenol content in crude rRCP ranged from 0.31% to 2.26%, ranked as HY > ZG > HF > FJ > WF > LC > XK > WS > JKH. HY exhibited significantly higher polyphenol content than the other regions. In the crude wRCP, the polyphenol content ranged from 0.32% to 4.25%, with HY and HF significantly higher than other regions. Notably, the polyphenol content of HY increased by 88.1% after processing (Figure 1C).

#### 3.1.2. In Vitro Antioxidant Activity of Crude RCP

**Figure 2:**
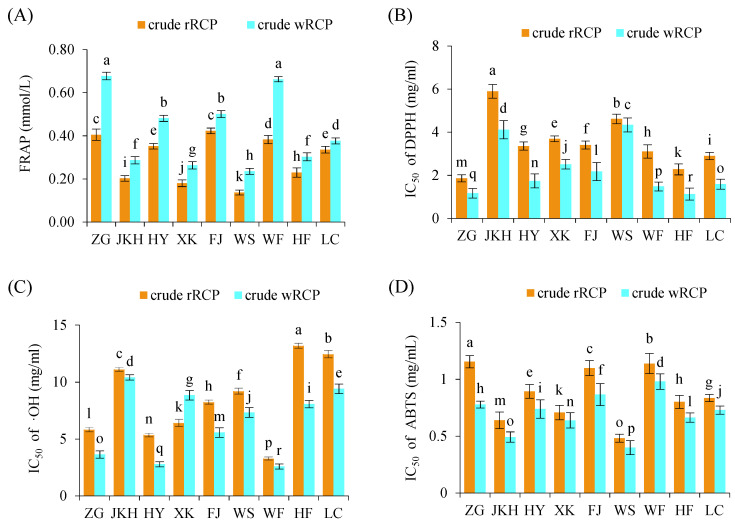
In vitro antioxidant activity of crude RCP. (**A**) Total antioxidant capacity (FRAP) of crude RCP; (**B**) IC_50_ value of DPPH radical scavenging activity; (**C**) IC_50_ value of ABTS radical scavenging activity. (**D**) IC_50_ value of hydroxyl radical (•OH) scavenging activity. Different letters (a–r) above the bars indicate statistically significant differences at *p* < 0.05.

The FRAP values of crude rRCP ranged from 0.14 to 0.42 mg/mL across regions, with FJ and ZG showing the strongest antioxidant capacities. Crude wRCP had FRAP values ranging from 0.24 to 0.68 mg/mL, with ZG and WF showing the highest values. In general, crude wRCP exhibited significantly stronger total antioxidant activities compared to crude rRCP (Figure 2A). The IC_50_ values for DPPH radical scavenging showed that ZG had the lowest IC_50_ value (1.86 mg/mL) among crude rRCP, while JKH had the highest (5.90 mg/mL). For crude wRCP, HF exhibited the strongest DPPH scavenging ability (IC_50_ exhibited the lowest). Notably, crude wRCP universally enhanced antioxidant activity, as evidenced by the significantly lower IC_50_ values compared to crude rRCP across all regions (Figure 2B). In ABTS radical scavenging, WS samples showed the highest scavenging activity (lowest IC_50_) among both crude rRCP and crude wRCP (Figure 2C). The IC_50_ values for •OH scavenging showed that the WF samples had the strongest scavenging ability in both crude rRCP and crude wRCP (Figure 2D). Overall, crude wRCP significantly enhanced the FRAP value and scavenging ability, as evidenced by the concomitant reductions in IC_50_ values for DPPH, ABTS, and •OH scavenging compared to crude rRCP.

#### 3.1.3. Principal Component Analysis (PCA)

The Kaiser–Meyer–Olkin (KMO) value was 0.701, indicating that PCA was suitable for the dataset (Table S1). PCA was applied to analyze five chemical components of *Radix Cyathula officinalis* Kuan samples from different regions before and after being wine-processed. Two principal components were extracted based on the criterion of eigenvalues greater than 1. These two components explained 71.975% of the total variance, indicating that they captured the majority of the information contained in the original dataset (Table 1). Principal Component 1 had the highest eigenvalue of 2.550, accounting for the largest proportion of variance (51.001%). Principal Component 2 had the second-highest eigenvalue of 1.049, with a variance contribution of 20.974%. Starting from the third component, the eigenvalue dropped significantly to 0.626, which is far below 1. Therefore, the first two components were selected as the principal components representing the five variables.

Based on the absolute value criterion (≥0.5), the component loadings with absolute values greater than 0.5 were ranked in descending order to obtain the final component matrix (Table 2). The first principal component (PC1) was mainly related to FRAP (0.910) and ABTS (0.708) activities, while the second principal component (PC2) was primarily associated with polysaccharide content (0.967). Notably, both FRAP and polysaccharide content had loading values greater than 0.9, suggesting that these two variables accounted for the majority of the information within the dataset.

Based on the component score coefficients, two principal component score functions were constructed as follows:

(1) Y_1_ = C_1_·X_1_ + C_2_·X_2_ + C_3_·X_3_ + C_4_·X_4_ + C_5_·X_5_

(2) Y_2_ = C_21_·X_1_ + C_22_·X_2_ + C_23_·X_3_ + C_24_·X_4_ + C_25_·X_5_

where C_i_ represents the component score coefficient/ √square root of the eigenvalue of the corresponding principal component; Y_1_ and Y_2_ represent the scores of PC1 and PC2, respectively; and X_1_–X_5_ correspond to the values of FRAP, DPPH, •OH, ABTS, and polysaccharide content.

A comprehensive evaluation model of antioxidant activity for *C. officinalis* polysaccharides was then constructed using a weighted combination of Y_1_ and Y_2_. The weights were assigned based on the proportion of each principal component’s variance contribution relative to the cumulative contribution of the two components. The composite scoring function was defined as follows:

(3) Y = Y_1_·S_1_ + Y_2_·S_2_

where Sᵢ represents the variance contribution rate of each principal component, and Y is the comprehensive score.

Using this model, the antioxidant activity scores for crude RCP from different regions were calculated (Table 3). The results showed that among all regions, the crude rRCP and crude wRCP from the ZG region had the highest comprehensive antioxidant activity scores, with values of 1.209 and 2.103, respectively. This indicates that the crude RCP from the ZG region possessed the strongest overall antioxidant capacity. Therefore, the crude rRCP and crude wRCP from the ZG region were further purified by dialysis using a cut-off membrane with 3500 Da molecular weight to obtain rRCP and wRCP for subsequent in vivo antioxidant activity studies.

#### 3.1.4. Component Composition and Fractionation of RCP

The composition of RCP showed that rRCP contains 89.55% polysaccharides, 1.59% proteins, and 1.03% polyphenols, while wRCP contains 91.26% polysaccharides, 1.28% proteins, and 0.86% polyphenols (Figure S1). Polysaccharides were the predominant components in both rRCP and wRCP, with no significant difference observed between the two (*p* < 0.05). The further fractionation of RCP using DEAE–agarose gel FF chromatography indicated that RCP solely consists of one homogeneous neutral polysaccharide and one homogeneous acidic polysaccharide (Figure S2). Specifically, from 500 mg of rRCP, 326.6 mg of neutral polysaccharides (65.32%) and 114.8 mg of acidic polysaccharides (22.96%) were obtained. Similarly, from 500 mg of wRCP, 366.5 mg of neutral polysaccharides (73.30%) and 84.3 mg of acidic polysaccharides (16.86%) were isolated. In both cases, neutral polysaccharides were the predominant fraction.

### 3.2. In Vivo Antioxidant Activities of RCP in C. elegans

#### 3.2.1. Effects on Lifespan and Reproduction

**Figure 3:**
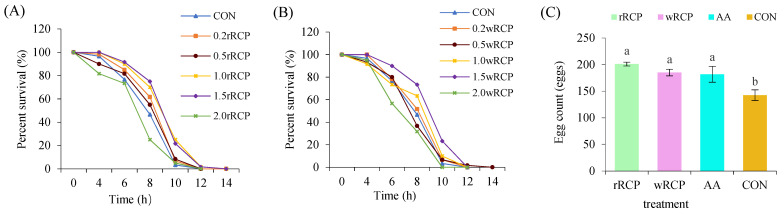
Effects of RCP on lifespan and reproduction in *C. elegans*. (**A**) Survival curves of *C. elegans* exposed to rRCP under heat stress; (**B**) survival curves of *C. elegans* exposed to wRCP under heat stress; (**C**) egg-laying performance of *C. elegans* treated with RCP. Different letters (a, b) above the bars indicate statistically significant differences at *p* < 0.05.

To efficiently assess the anti-aging and stress resistance abilities of rRCP and wRCP, we established appropriate concentrations (0.2–2.0 mg/mL) of the polysaccharides to maintain *C. elegans*. Under various concentrations of rRCP and wRCP treatments, the apoptosis trends of *C. elegans* were consistent, showing a deceleration at 0.2, 0.5, 1.0, and 1.5 mg/mL, but a slight acceleration at 2.0 mg/mL; however, the difference was not statistically significant (*p* < 0.05). The *C. elegans* treated with rRCP and wRCP at 1.5 mg/mL exhibited the longest survival under thermal stress (35 °C), with survival significantly prolonged compared to the control (CON) group (Figure 3A,B). The reproductive capacity of *C. elegans* was evaluated based on egg-laying numbers. The number of eggs laid under different treatments followed the order rRCP > wRCP > AA > CON. The egg-laying numbers in the rRCP, wRCP, and AA groups were significantly higher than those in the CON group (*p* < 0.05), while no significant differences were observed among the rRCP, wRCP, and AA groups. These results indicate that the egg-laying of *C. elegans* was significantly enhanced by both rRCP and wRCP treatments, with no signs of reproductive toxicity (Figure 3C).

#### 3.2.2. Effects of rRCP and wRCP on Antioxidant Enzyme Activities in *C. elegans*

**Figure 4:**
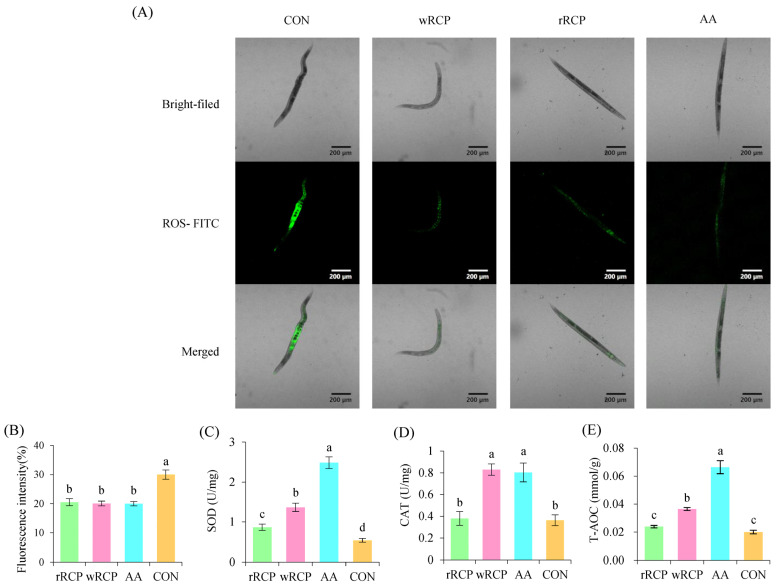
Effect of RCP on antioxidant enzymes activities in *C. elegans*. (**A**) Intracellular ROS fluorescence in *C. elegans* after heat stress, visualized by fluorescence microscopy using an ROS-specific probe. (**B**) Effect of RCP on ROS levels in *C. elegans* after heat stress. (**C**) Effect of RCP on SOD activity in *C. elegans* after heat stress. (**D**) Effect of RCP on CAT capacity in *C. elegans* after heat stress. (**E**) Effect of RCP on T-AOC in *C. elegans* after heat stress. Different letters (a–d) above the bars indicate statistically significant differences at *p* < 0.05.

Thermal stress, a form of intrinsic cellular stress, can lead to significant ROS accumulation in cells. To investigate the effects of rRCP and wRCP on the antioxidant defense system in *C. elegans*, we measured the activities of antioxidant enzymes and total antioxidant capacity (T-AOC) in *C. elegans* treated with rRCP and wRCP. Compared to the CON group, rRCP, wRCP, and AA treatments significantly reduced the fluorescence intensity of ROS in *C. elegans*, with wRCP showing slightly stronger effects (Figure 4A,B). The fluorescence intensity results revealed that the ROS levels of the rRCP, wRCP, and AA groups decreased by 31.67%, 33.14%, and 33.37%, respectively.

SOD and T-AOC activities were significantly enhanced in the rRCP, wRCP, and AA groups compared to the CON group. In *C. elegans* pretreated with rRCP, wRCP, and AA, SOD activities significantly increased by 42.37%, 63.92%, and 80.79%, respectively (Figure 4C). Meanwhile, T-AOC levels in the rRCP, wRCP, and AA treatment groups of nematodes increased by 16.25%, 45.23%, and 69.9% compared to the CON group (Figure 4E). The CAT activities in the *C. elegans* treated with wRCP and AA significantly increased, by 56.23% and 54.77%, respectively (Figure 4D). However, no significant difference was observed in CAT activity between the rRCP and CON groups. These results suggest that the wRCP group showed higher antioxidant activity compared to the rRCP group.

### 3.3. Effect of RCP on Gut Microbiota of C. elegans

#### 3.3.1. Effect of RCP on Microbial Diversity

**Figure 5:**
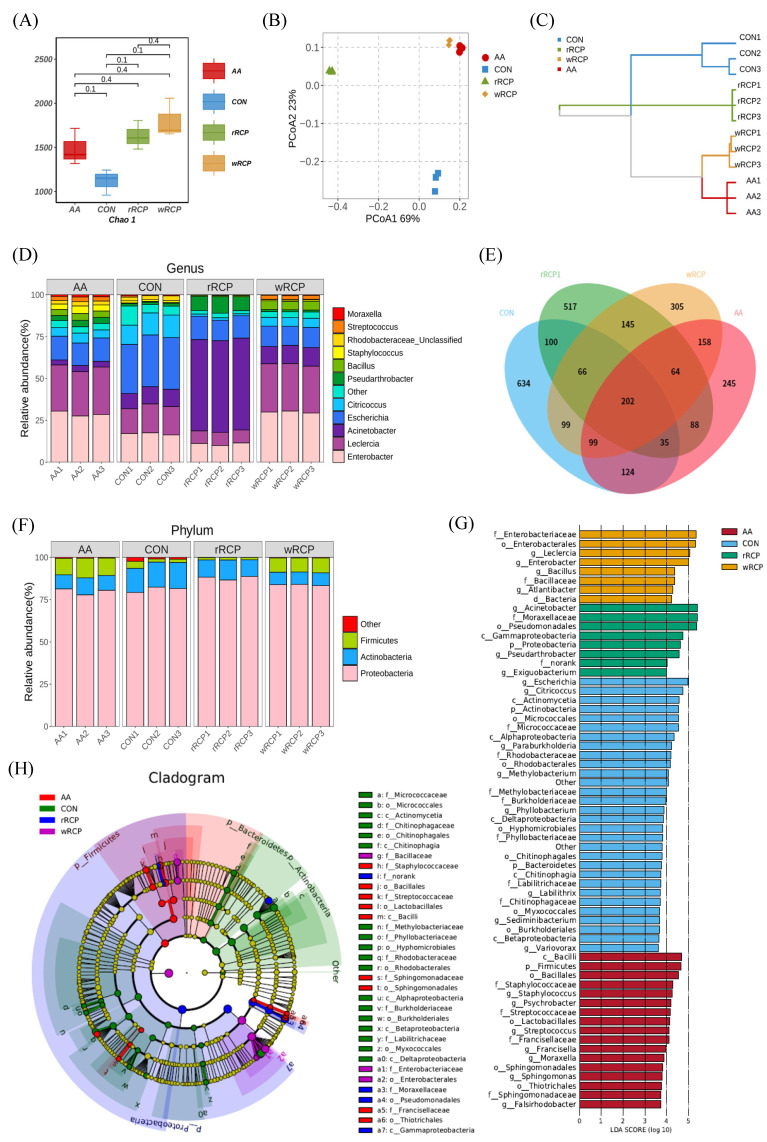
Effects of RCP on gut microbiota structure in *C. elegans*. (**A**) Chao 1 index of microbial diversity. (**B**) PCoA of bacterial community composition at the OTU level. (**C**) Hierarchical clustering analysis. (**D**) Relative abundance of microbiota at the genus level. (**E**) Venn diagram showing shared and unique OTUs across groups. (**F**) Relative abundance of microbiota at the phylum level. (**G**) LDA score plot based on LEfSe analysis. Enriched taxa with an LDA score >3.6 are shown in the histogram. (**H**) LEfSe cladogram highlighting differentially abundant taxa. Colors represent different groups, and colored circles indicate biomarkers. From the inner to outer circles, taxonomic levels range from phylum to genus. The circle size corresponds to taxon abundance.

To explore the effect of RCP on the gut microbiota, we analyzed the diversity of the gut microbiota using high-throughput sequencing. Venn diagram analysis at the OTU level showed that 202 OTUs overlapped among the four groups, with a cut-off condition of 97% sequence similarity. The CON group had 634 unique OTUs, the AA group had 245, the wRCP group had 305, and the rRCP group had 517 (Figure 5E). The gut microbiota diversity was assessed using alpha diversity analysis, and the results showed that the Chao 1 index for the rRCP, wRCP, and AA groups was higher than that of the CON group, indicating that the CON group had the lowest species diversity. These results suggest that the RCP treatment significantly altered the diversity of the gut microbiota (Figure 5A). To visualize the dispersion and aggregation of the microbiota among the groups, OTU principal coordinate analysis (PCoA) and hierarchical clustering analysis, respectively, were employed to observe the similarities and differences between them. The top two principal components in PCoA accounted for 69% and 23% of the total data, respectively (Figure 5B). The results showed that the species distribution in the rRCP group was distinct from the CON group, while the wRCP and AA groups were clustered together. Similar observations were obtained in hierarchical clustering analysis (Figure 5C). These findings suggest that rRCP and wRCP can modify the composition and diversity of the gut microbiota, with the wRCP group having a more pronounced effect. Its impact on the microbial community’s composition and diversity was comparable to that of the AA group.

#### 3.3.2. Effect of RCP on Gut Microbiota Composition and Structure

To further investigate the specific changes in microbial communities, we analyzed the taxonomic composition of each group’s samples at the phylum level. *Proteobacteria* dominated, with over 75% relative abundance, followed by *Actinobacteria* and *Firmicutes*. Compared with the CON group, the abundance of *Actinobacteria* decreased in the rRCP, wRCP, and AA groups. The abundance of *Firmicutes* increased in the AA and wRCP groups, while the abundance of *Proteobacteria* increased in the rRCP group (Figure 5F). At the genus level, *Enterobacter*, *Leclercia*, *Acinetobacter*, *Escherichia*, *Citricoccus*, *Pseudarthrobacter*, *Bacillus*, *Staphylococcus*, *Rhodobacteraceae_Unclassified*, *Streptococcus*, and *Moraxella* were identified as the 11 microbiota demonstrating relative abundances exceeding 1% (Figure 5D). Among these classified genus, the abundance of *Escherichia* was highest in the CON group, followed by *Leclercia* and *Enterobacter*. The AA and wRCP groups exhibited the highest relative abundances of *Leclercia* and *Enterobacter*, followed by *Escherichia*. In the rRCP group, *Acinetobacter* demonstrated the greatest abundance, with *Escherichia* and *Leclercia* ranking second and third, respectively. Compared with the CON group, the AA group exhibited reduced abundances of *Acinetobacter*, *Escherichia*, and *Citricoccus*, while *Rhodobacteraceae* (Unclassified) remained unchanged, and other bacterial genera showed increased abundance profiles. The rRCP group showed an increase in *Acinetobacter*; *Pseudarthrobacter*, and *Bacillus* were raised, while other bacterial genera decreased. The wRCP group exhibited a significant increase in the abundance of *Enterobacter*, *Leclercia*, *Bacillus*, and *Streptococcus*, while the abundance of *Acinetobacter* remained unchanged. All other bacterial genera demonstrated a marked reduction in their abundances. Compared with the CON group, the AA, wRCP, and rRCP groups showed reduced abundances of *Escherichia* and *Citricoccus*, along with an increased abundance of *Bacillus*.

A linear discriminant analysis (LDA) effect size (LEfSe) was applied to identify key phylotypes of the gut microbiota in different groups. The LDA results (Figure 5G) showed 28 discriminative features in the CON group (LDA > 3.6, *p* < 0.05), including 5 Actinobacteria, 5 Bacteroidetes, and 18 Proteobacteria at the phylum level. The predominant microbial taxa were identified as *Escherichia*, *Citricoccus*, and *Actinomycetia*. The wRCP group also showed eight dominant microorganisms (LDA > 3.6, *p* < 0.05), including two Firmicutes and five Proteobacteria at the phylum level. The major microbiota were *Enterobacteriaceae*, *Enterobacterales*, and *Leclercia*. The rRCP group exhibited eight discriminative features (LDA > 3.6, *p* < 0.05), comprising one Actinobacterium, one Firmicute, and five Proteobacteria at the phylum level. The major microbiota were *Acinetobacter*, *Moraxellaceae* and *Pseudomonadales*. The AA group displayed 17 discriminative features (LDA > 3.6, *p* < 0.05), comprising 8 Firmicutes and 9 Proteobacteria at the phylum level. The predominant microbial taxa were identified as *Bacilli*, *Firmicutes*, and *Bacillales*. An evolutionary clustering analysis diagram was then constructed to identify major microflora by taxonomy (Figure 5H). In the cladogram, *Firmicutes* and *Proteobacteria* had the highest abundance in the AA group (red parts), while *Proteobacteria* had the highest abundance in the wRCP group (purple parts) and rRCP group (blue parts), followed by *Firmicutes*. In the CON group (green parts), *Proteobacteria* exhibited the highest relative abundance, while *Bacteroidetes* represented a unique microbial signature specific to this group. Compared to the CON group, the wRCP, rRCP, and AA groups exhibited a reduction in *Bacteroidetes* and a significant increase in *Firmicutes*. Additionally, the wRCP and rRCP groups showed an increase in the *Bacillales* order within *Firmicutes*, while the AA group showed increases in both the *Bacillales* and *Lactobacillales* orders with the same phylum. Overall, these results indicate that the RCP treatment altered the key phylotypes of gut microbiota in *C. elegans* and promoted the multiplication of specific bacteria.

## 4. Discussion

In recent years, it has been widely recognized that the oxidative stress induced by excessive ROS is closely associated with the pathogenesis of neurodegenerative diseases [32], representing one of the major mechanisms underlying neuronal damage and death. Antioxidant interventions have been shown to alleviate oxidative stress and improve symptoms in various neurodegenerative models, making the development of antioxidant-based therapies a promising strategy [33,34].

Polysaccharides derived from medicinal plants have been attracting increasing attention as potential natural antioxidants due to their strong antioxidant activities and low cytotoxicity [35,36,37]. In this study, crude RCP was extracted from the roots of *Radix Cyathula officinalis* Kuan, with yields ranging from 29.77% to 43.42%, consistent with the ranges reported by Zhao Yueqi [38], Ye Xiujuan [39], and Wang Yuanyuan et al. [40]. Significant differences were observed in polysaccharide content among different production regions and between crude rRCP and crude wRCP, consistent with findings by Lai Xin [41], confirming that both region and processing method affect polysaccharide content. In vitro antioxidant assays revealed substantial variations in antioxidant capacities among samples from different regions and between crude rRCP and crude wRCP, aligning with the findings of Chen Aimeng et al. [42]. Generally, crude wRCP exhibited significantly higher antioxidant capacity compared to crude rRCP. Considering the protein and polyphenol contents, this is likely attributed to the increased polyphenol content. A similar phenomenon was observed by Zdenka Hromádková et al. [43] in their study on the antioxidant activity of wheat bran polysaccharides, in which higher polyphenol content was associated with stronger hydroxyl radical scavenging activity.

To further explore the in vivo antioxidant capacity and underlying mechanisms of RCP, we selected the crude RCP extracted from ZG samples, which exhibited the highest comprehensive antioxidant score based on PCA. We found that purified RCP comprises a single homogeneous neutral polysaccharide and a single homogeneous acidic polysaccharide, with neutral polysaccharides being the predominant component in both extracts. Similar findings have been reported in *Allium macrostemon* Bunge [44], *Epimedium acuminatum* Franch. [45], and *Polygonum multiflorum* Thunb [46]. However, no such results have been reported for *Cyathula officinalis* Kuan to date. This novel finding establishes a critical scientific basis for further experimental research and analysis.

**Figure 6:**
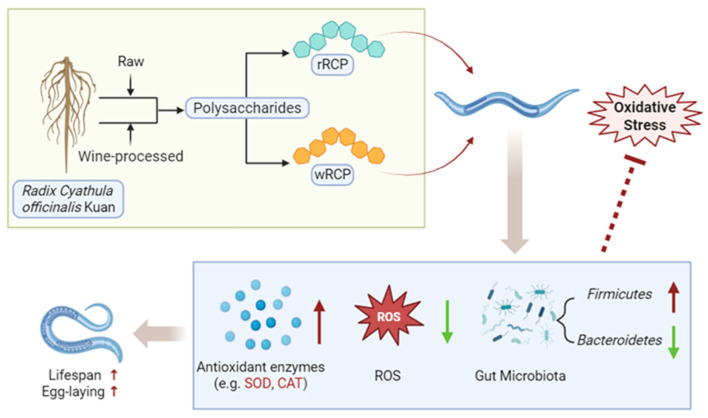
Schematic representation the underlying mechanism of antioxidant activity of RCP. The red upward arrows represent an increase, whereas the green downward arrows represent a decrease.

To further investigate the antioxidant effects of RCP, we evaluated the in vivo antioxidant effects of RCP under heat stress using a *C. elegans* oxidative stress model. Survival analysis of this study showed that RCP treatments at concentrations of 0.2, 0.5, 1.0, and 1.5 mg/mL all extended the survival rate of *C. elegans*, with the most pronounced effect observed at 1.5 mg/mL. However, treatment with RCP at 2.0 mg/mL resulted in a slightly lower survival rate compared to the CON group, although the difference was not statistically significant. A similar trend was observed in a study by Li Meilin et al. [47], in which *C. elegans* treated with *Cornus officinalis* polysaccharides exhibited a comparable dose-dependent pattern. Comparable biphasic responses have also been reported in lifespan studies of *C. elegans* treated with fructose [48], folic acid [49], and epigallocatechin-3-gallate [50]. Furthermore, we confirmed that RCP prolongs the lifespan without impairing the reproductive function of *C. elegans*. In vivo antioxidant assays showed that both rRCP and wRCP significantly enhanced the antioxidant defense system of *C. elegans*, reduced ROS accumulation, and increased the activity of key antioxidant enzymes such as SOD and CAT, with wRCP exhibiting a more pronounced effect than rRCP (*p* < 0.05). Similarly, Hou Shoubu et al. [51] reported that different processing methods (Paozhi) influenced the antioxidant activity of polysaccharides from *Cistanche deserticola*. Specifically, stir-baking with wine altered the structure of the polysaccharides, resulting in significantly higher antioxidant activity, suggesting that the structural modifications induced by processing may enhance the antioxidant properties of polysaccharides. In summary, the above findings suggest that RCP may extend the lifespan of *C. elegans* within an optimal concentration range by modulating antioxidant enzyme activity and reducing oxidative damage (Figure 6).

Previous studies have shown that oxidative stress is closely associated with the gut microbiota, and that gut microbial communities play a crucial role in the interactions between polysaccharides and the host. Polysaccharides may either be degraded into smaller absorbable molecules or modulate host health by reshaping gut microbiota composition [52,53,54,55]. In this study, rRCP and wRCP reduced the relative abundance of *Bacteroidetes* and *Actinobacteria* while increasing the *Firmicutes* in *C. elegans*. Notably, the changes in the wRCP group were similar to those in the AA group. Similar microbial shifts have been observed in experiments with polysaccharide-treated mice [56,57], human colitis patients [58,59], traumatic brain injury models [60], and patients with Alzheimer’s disease [61]. Numerous studies suggest that ecological dysbiosis is typically characterized by an elevated abundance of *Bacteroidetes* and *Proteobacteria*, coupled with a reduction in *Firmicutes* [62,63]. Additionally, studies have demonstrated that, with advancing age, the abundance of *Bacteroidetes* and *Proteobacteria* increases, while that of *Firmicutes* decreases [64]. These findings suggest that RCP may exert effects via gut microbiota modulation, specifically by increasing *Firmicutes* and decreasing *Bacteroidetes* during host–microbiota interactions, thereby extending the lifespan of nematodes (Figure 6).

Numerous studies have found that, at the genus level, RCP treatment induced changes in functional bacterial genera, including reductions in *Escherichia* and *Citricoccus* and increases in *Bacillus*, *Streptococcus*, *Pseudarthrobacter*, *Enterobacter*, *Streptococcus*, and *Leclercia*. For example, increased levels of *Escherichia*/*Shigella* have been observed in cognitively impaired older adults [65], while polysaccharide treatments with tea [66], Grifola frondosa [67], and Paeoniae *Radix* Alba [68] have been shown to reduce harmful genera and promote beneficial ones. Consistent with these reports, both rRCP and wRCP significantly reduced *Escherichia* and *Citricoccus*, while increasing *Bacillus* and *Streptococcus*. Specifically, the AA group showed an increase in both *Bacillales* and *Lactobacillales*. Extensive studies have revealed that *Lactobacillus* species possess important biological properties, including the secretion of antimicrobial substances, the improvement of the intestinal environment, and the maintenance of gut microbiota homeostasis [69,70]. Similarly, *Bacillus* species are common intestinal probiotics that have demonstrated significant potential in alleviating gut microbiota dysbiosis [71,72]. For example, *Bacillus licheniformis* and *Bacillus subtilis* have been shown to significantly reduce intracellular ROS levels [73]. *Bacillus* spp. mitigate oxidative stress through the action of SOD, which converts excess superoxide anions into hydrogen peroxide [74]. In conjunction with the significant increase in SOD enzyme activity observed in this study, we hypothesize that RCP treatment enhances the abundance of beneficial *Firmicutes*, especially *Bacillus*, which counteract oxidative stress through elevated SOD activity.

Multiple studies have identified that polysaccharides extracted from *Cyathula officinalis* and *Achyranthes bidentata* exhibit similar structural characteristics, primarily being composed of fructose and glucose in an approximate ratio of 20:1, with fructose the predominant monosaccharide, comprising 70–80% of the composition [22,75,76,77,78]. Previous research indicates that fructose is resistant to hydrolysis by human digestive enzymes, but can be fermented by the gut microbiota to produce short-chain fatty acids (SCFAs). Hua-Yang Si et al. [79] demonstrated that polysaccharides extracted from *Achyranthes bidentata* are degraded by the gut microbiota into SCFAs in mice. Similarly, Chang Wen et al. [80] reported analogous findings with polysaccharides from *Achyranthes bidentata*. It is well-known that *Firmicutes* are the main producers of SCFAs in the intestine, as they are the main degraders of indigestible polysaccharides. Based on these observations, we hypothesize that RCP is likely to modulate gut microbiota composition by promoting the production of SCFAs. However, the specific underlying mechanisms require further investigation.

Collectively, our findings indicate that the antioxidant effects of RCP are mediated through both the direct enhancement of endogenous antioxidant defenses and the modulation of the gut microbiota, which together contribute to mitigating oxidative damage. Further studies are needed to elucidate the precise molecular pathways, especially concerning interactions with host signaling and microbial metabolites.

Although this study utilized the *C. elegans* model, this organism has been well-established to exhibit 60–80% genetic homology with human genes. Numerous comparative studies have demonstrated a significant degree of conservation in the antioxidant and anti-aging regulatory mechanisms of natural antioxidants across *C. elegans*, mammals, and humans. The antioxidant and anti-aging effects observed in the *C. elegans* model can be translated to clinical practice, which will reflect a broader spectrum of aging-related characteristics and diseases [81,82]. Our findings provide a valuable reference for the development and application of RCP in mammals and humans.

## 5. Conclusions

In this study, a crude RCP was extracted from *Radix Cyathula officinalis* Kuan, exhibiting significant in vitro antioxidant capacity. Both polysaccharide content and antioxidant capacity varied significantly depending on the geographic region and processing method, with wine-processed RCP significantly enhancing the antioxidant potential. Principal component analysis identified ZG samples as having the highest antioxidant potential. Subsequently, the crude rRCP and wRCP from this region were further investigated and assessed using a *C. elegans* oxidative stress model. Both rRCP and wRCP significantly enhanced antioxidant enzyme activities, reduced ROS levels, and extended the lifespan of *C. elegans* under heat stress. Notably, wRCP exhibited stronger antioxidant effects, suggesting that wine-processing may enhance RCP’s bioactivity. Additionally, both treatments modulated the gut microbiota, increasing microbial diversity, enriching beneficial bacteria, and reducing harmful genera. These microbial changes likely contributed to the observed antioxidant benefits and lifespan extension. Overall, our findings provide a theoretical basis for the further development and application of RCP. Moreover, this study highlights the importance of the production region and processing method in optimizing the bioactivity of polysaccharides from *Radix Cyathula officinalis* Kuan. Nevertheless, further investigation is needed to elucidate the detailed antioxidative mechanisms.

## Figures and Tables

**Table 1:** Explanation of total variance.

Component	Initial Eigenvalues				Extraction Sums of Squared Loadings	
	Total	% of variance	Cumulative %	Total	% of variance	Cumulative %
1	2.550	51.001	51.001	2.550	51.001	51.001
2	1.049	20.974	71.975	1.049	20.974	71.975
3	0.626	12.527	84.502			
4	0.547	10.931	95.433			
5	0.228	4.567	100.000			

**Table 2:** Component matrix ^a^.

	Constituent	
	1	2
FRAP	0.910	−0.061
DPPH	−0.786	−0.217
•OH	−0.772	0.250
ABTS	0.708	−0.012
Polysaccharide content	0.090	0.967

The letter ‘^a^’ represents the rotated factor loading matrix.

**Table 3:** Comprehensive scores of antioxidant activity of crude RCP.

	Regions	Comprehensive Score (Y)
Crude rRCP	ZG	1.209
	HY	1.191
	WF	0.512
	FJ	0.242
	HF	−0.159
	LC	−0.458
	XK	−0.543
	JKH	−1.86
	WS	−2.431
Crude wRCP	ZG	2.103
	HY	2.088
	WF	2.037
	FJ	1.344
	HF	0.657
	LC	−0.26
	XK	−0.597
	JKH	−1.767
	WS	−2.309

Higher scores indicate stronger overall antioxidant capacity.

## Data Availability

The original contributions presented in this study are available in the article or Supplementary Materials.

## Supplementary Materials

The following supporting information can be downloaded at https://www.mdpi.com/article/10.3390/cimb47070538/s1.
