# Supplementary material for: Antioxidant Activity of Radix Cyathula officinalis Kuan Polysaccharides and Their Modulatory Effects on the Gut Microbiota of Caenorhabditis elegans
Source: Curr Issues Mol Biol. 2025 Jul 11;47(7):538. doi: 10.3390/cimb47070538 (PMC12293607; doi:10.3390/cimb47070538)
Supplement: Supplementary file 1 [file cimb-47-00538-s001.zip › Supplementary Materials.pdf]

Antioxidant Activity of *Radix Cyathula officinalis* Kuan Polysaccharides and Their Modulatory Effects on the Gut Microbiota of *Caenorhabditis elegans*

Rui Li [1,2], Xinyue Chen [1], Lijuan Wu [1], Lei Xie [1], Mengqiu Chen [1], Yujie Qiu [1], Fan Liu [1], and Ji Chen [1,*] and Mengliang Tian [1,*]

[1] College of Agronomy, Sichuan Agricultural University, Chengdu 611130, China; 71352@sicau.edu.cn (R.L.); chenxinyue@tfswufe.edu.cn (X.C.); wulijuan0921@126.com (L.W.); 17683180748@163.com (L.X.); 19114323880@163.com(M.C.); 80254@sicau.edu.cn (Y.Q.); Liufantl2006@163.com (F.L.).

[2] Academy of Agriculture and Forestry Sciences, Qinghai University, Xining, China 810016, China.

* Correspondence: jichen@sicau.edu.cn (J.C.); secondat@sicau.edu.cn (M.T.)

Supplementary data:

Table S1. KMO and Bartlett test.

**KMO Measure of Sampling Adequacy**		0.701
Bartlett's Test of Sphericity	χ^2	22.696
	df	10
	p	0.012

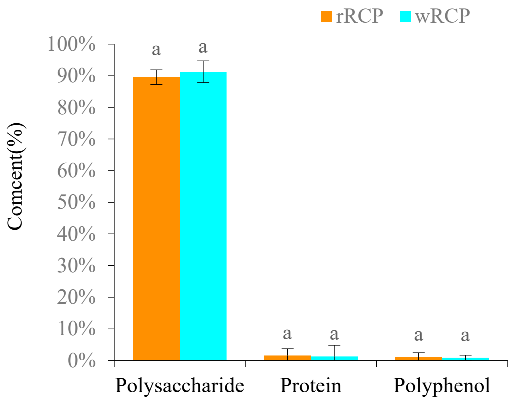

Figure S1 Composition of RCP

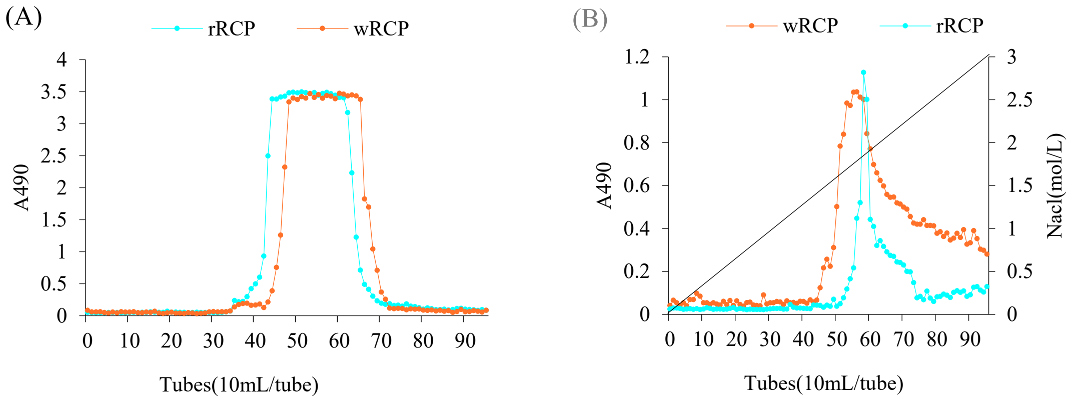

Figure S2 Elution profile of RCP. (A) Elution profile of neutral polysaccharides. (B) Elution profile of acidic polysaccharides.
